# Biology, Immunology, Epidemiology, and Therapy of Fungal Infections: A Themed Issue Dedicated to Professor David A. Stevens: Editorial

**DOI:** 10.3390/jof11030179

**Published:** 2025-02-25

**Authors:** Raquel Sabino, David A. Stevens

**Affiliations:** 1 Faculdade de Farmácia, Universidade de Lisboa, 1649-003 Lisbon, Portugal; 2 Faculdade de Medicina, Instituto de Saúde Ambiental, Universidade de Lisboa, 1649-028 Lisbon, Portugal; 3 Laboratório Associado TERRA—Laboratório para o Uso Sustentável da Terra e dos Serviços dos Ecossistemas, Instituto Superior de Agronomia, 1349-017 Lisbon, Portugal; 4 Division of Infectious Diseases and Geographic Medicine, Stanford University Medical School, Stanford, CA 94305, USA; stevens@stanford.edu

## 1. Introduction

The fungi command our attention, as there are >5 million species alive sharing our planet today, and they are plant pathogens resulting in up to 50% of crop losses in some countries [1,2,3]. The fungal kingdom is versatile, and members exist in water and in terrestrial environments, under varying temperature and moisture conditions. The importance of *medical* mycology has risen owing to the steady increase in the number and severity of fungal infections, the wide diversity of etiological agents, new environmental conditions of the human host, and increasing environmental exposure to fungi.

There has been a marked increase in invasive fungal infections, over several decades, with 3.75 million people dying annually presently [4] from fungal infections. The increased use of immunosuppression to treat a variety of diseases, including the increased use of transplantation to treat organ dysfunction, and more aggressive treatment of malignancies, and the successes of such treatments, leads to more patients surviving in an immunosuppressed state. These patients are at risk of fungal infections, as fungi can be adept opportunists, especially when bacterial competitors are reduced by the advances in antibacterial therapy. Successes in the treatment of other, nonmalignant morbidities (e.g., cystic fibrosis, bronchiectasis) have reduced their mortality, but not eliminated the risk factors which continue to expose the surviving patients to fungal infections. The burden of fungal infections is augmented by the fungal etiology of ocular pathology and blindness, debilitating allergies, recurrent infections (e.g., vulvovaginal candidiasis), nosocomial infections, other serious but nonfatal conditions, and mycoses of domesticated animals. Fungi have also been conjectured as possible bioweapons in the future [2].

The development of antifungal drugs is difficult, because we animals share many potential drug targets with our fellow eukaryotes, the fungi. The emergence of antifungal resistance has also contributed to the persistence of fungal infections and to the growing number of deaths [5,6,7,8,9].

At the same time, climate change, particularly global warming and extreme weather events, is also causing shifts in the ecological niches of many environmental saprophytic fungi [10], leading to the emergence of new pathogenic agents, such as the highly resistant *Candida auris* strains [11]. In addition, the identification of endemic fungal pathogens in newer geographical locations and environmental fungal pathogens in extended environments, both also related in part to climate change and to changing industrial and agricultural practices, has also highlighted the importance of comprehensively addressing human fungal diseases from a One Health perspective [12,13].

In 2022, the World Health Organization (WHO) published the first fungal priority pathogens list, spotlighting 19 fungal pathogens, with the aim of focusing research and policy interventions on strengthening the global response to fungal disease, especially for those agents [14]. This action aimed to improve patient outcomes, guide public health interventions, and address the challenges posed by fungal infections in a changing world, which are often underestimated but pose significant health risks globally.

Presently, research on mycoses amounts to <1.5% of all infectious disease research funding [14]. An increased awareness of these infections is, therefore, of utmost importance to develop a global response to the complex challenges, integrating scientific research, public health, and political efforts to mitigate the harmful effects of fungal infections.

This Special Issue aims to address the issues outlined above. Hence, the most recent trends in epidemiology, antifungal resistances, diagnostics, risk factors, immunology, and therapeutic approaches relevant to fungal infections are discussed with mastery by prominent scientists of this field.

## 2. A Synopsis of the Key Papers in This Special Issue

### 2.1. Epidemiology

Understanding the epidemiology of fungal agents and their antifungal resistances is essential in public health, clinical medicine, and research. Better diagnostic protocols, antifungal stewardship, and deeper research to increase knowledge in this area are needed to mitigate the impact of invasive fungal infections. The following four papers [Contributed Papers 1–4] address these issues:

**Antifungal Resistance and Genotyping of Clinical *Candida parapsilosis* Complex in Japan [Contributed Paper 1].**

The *C. parapsilosis* complex has become a major contributor to invasive fungal infections globally. Concerns over antifungal resistance necessitate studies to monitor susceptibility trends and understand resistance mechanisms. Khalifa and co-authors analyzed, including genotyping and molecular analyses for resistance, *C. parapsilosis* clinical isolates from 76 hospitalized patients in Japan (2005–2019), where most originated from blood. All isolates were susceptible to the tested antifungal agents, and no mutations linked to resistance were detected. This study highlights the susceptibility of *C. parapsilosis* complex isolates in Japan to current antifungal agents, with no evidence of resistance-associated mutations. Microsatellite typing suggested limited diversity among isolates. These findings provide a baseline for future surveillance and emphasize the importance of monitoring resistance trends to ensure effective treatment strategies.

**Coccidioides Species: A Review of Basic Research: 2022 [Contributed Paper 2]**

Theo Kirkland has assembled a bevy of eminent scientists, including experts in basic and clinical mycology, and provided a comprehensive review of research on *Coccidioides*, which would enable any reader to grasp the entire spectrum of information on the mycology of Coccidioides, gathered over decades. This review underscores significant strides in Coccidioides research, highlighting its complex lifecycle, ecological niche, and antigenic properties. These insights provide a foundation for improving diagnostics, treatment, and public health responses to this increasingly relevant pathogen. Despite advances, the biological mechanisms underlying spherule differentiation and immune evasion are unclear. Further studies are needed to map phenotypic differences between *C. immitis* and *C. posadasii*, elucidate host–pathogen interactions, and refine environmental detection methods.

**Epidemiology of Coccidioidomycosis in the Veterans Health Administration, 2013–2022 [Contributed Paper 3]**

Lucero-Obusan and co-authors performed a retrospective study analyzing electronic health records of the U.S. Veterans Health Administration (VHA) population (67,526 veterans). In the study, the 4204 positive samples obtained by culture or serology underscore the importance of addressing racial and ethnic disparities in coccidioidomycosis incidence and severity. The geographic distribution aligns with known endemic areas, but environmental and occupational exposures, particularly among veterans, likely contribute to the disease burden. Despite increased testing, significant delays in diagnosis remain a concern, exacerbating risks for severe outcomes. These data should also be useful in developing preventive strategies and improving the timing of treatment.

**Epidemiologic Aspects of Mycetoma in Africa [Contributed Paper 4]**

Develoux presents a review on the epidemiological aspects of mycetoma (classified as a neglected tropical disease by the WHO in 2016), emphasizing its chronic, disabling nature and its association with rural populations in arid tropical regions. These microbes are present in the environment, and infection results from trauma. The paper details the geographical distribution, environmental factors, etiological agents, and prevalence of mycetoma while highlighting diagnostic challenges and gaps in current knowledge. Mycetoma diagnosis remains challenging due to limited resources and inadequate diagnostic tools in endemic areas, and there is a critical need for more comprehensive field studies, species identification using molecular tools, and mapping of disease distribution. Recently developed molecular methods can be used to identify causal agents. Expanded research in underrepresented regions, alongside improved diagnostics, could lead to better disease management and prevention strategies.

### 2.2. Fungal Infections and COVID-19

CAPA (COVID-Associated Pulmonary Aspergillosis) is a recognized complication of severe COVID-19, often associated with invasive mechanical ventilation and immunosuppressive therapies. Its incidence varies globally, influenced by diagnostic approaches and healthcare resource availability. Early studies indicated mortality rates exceeding 50%, underscoring its clinical significance. In hospitalized and critically ill patients, *Candida* infections have also emerged as a concerning combination with COVID-19 infection. Understanding fungal and virus interaction is crucial for effective management and improving patients’ outcomes. The following three papers highlight the clinical relevance of infection with both SARS-CoV-2 and fungi [Contributed Papers 5–7]:

**Comparison of Multi-locus Genotypes Detected in Aspergillus fumigatus Isolated from COVID Associated Pulmonary Aspergillosis (CAPA) and from Other Clinical and Environmental Sources [Contributed Paper 5]**

Morais et al. investigated the genetic diversity of *A. fumigatus* isolates from CAPA patients and compared them with other clinical and environmental sources, studying their relationships by microsatellite genotyping. CAPA isolates exhibited high genetic heterogeneity, with no clustering or shared genotypes. However, from all isolates from the different sources tested, some genotypes were shared between clinical and environmental isolates, highlighting the importance of adopting a One Health perspective.

**Mortality in ICU Patients with COVID-19-Associated Pulmonary Aspergillosis [Contributed Paper 6]**

Aiming to evaluate ICU mortality trends in patients with CAPA, Beltrame and co-authors conducted a systematic review of 38 studies involving 1437 patients in several countries, highlighting factors influencing outcomes and gaps in management strategies. Mortality disparities highlight the need for standardized diagnostic protocols and timely intervention. Also, antifungal resistance, especially among azoles, poses an additional challenge in CAPA management. The authors pointed out that the rising incidence of CAPA parallels the widespread use of corticosteroids and prolonged ventilation in severe COVID-19 cases. There’s an urgent need to improve prevention and management strategies for CAPA, and research is needed to identify optimal treatment strategies to reduce mortality rates.

**Yeast Bloodstream Infections in the COVID-19 Patient: A Multicenter Italian Study (FiCoV Study) [Contributed Paper 7]**

Fungemia co-infection contributes to the severity of the condition of critically ill COVID-19 patients. The FiCoV study was conducted by Prigitano et al. in 10 Italian tertiary hospitals (February 2020–June 2021) and investigated the prevalence, risk factors, and outcomes of yeast bloodstream infections (BSIs) in hospitalized COVID-19 patients, focusing on antifungal resistance and mortality rates. Among 27,981 COVID-19 patients, 296 (1.06%) developed yeast BSIs. Regarding the etiological agent of infection, *Candida parapsilosis* was the most frequent (49.8%, with 72.6% fluconazole-resistant). The next most common were *Candida albicans* (35.2%, with 3.8% resistance to fluconazole) and *C. glabrata* (the nomenclature was updated for this microbe to *Nakaseomyces glabrata*) (10%, with 42.8% resistance to itraconazole). Antifungal treatment was given to 75.6% of patients, with echinocandins the primary choice. Although antifungal therapy improved outcomes, the mortality rate remained significantly higher than in patients without BSIs (45.5% versus 30.5%). The study emphasizes *Candida* infections in COVID-19 patients, emphasizing the identified species and highlighting the growing concern over antifungal resistance. Future research should further explore the modifiable risk factors and develop strategies to optimize patient outcomes in critically ill populations.

### 2.3. Novel Treatment Approaches

Current antifungals have limitations due to toxicity, drug interactions, and rising resistance, necessitating new therapeutic agents with novel mechanisms of action. This situation emphasizes the development of novel antifungal therapies to address rising resistance and improve outcomes in fungal infection management, as discussed in the following three papers [Contributed Papers 8–10]:

**Novel Antifungals and Aspergillus Section Terrei with Potpourri Susceptibility Profiles to Conventional Antifungals [Contributed Paper 8]**

The study developed by Vahedi-Shahandashti et al. thoroughly evaluates the in vitro efficacy of four novel antifungal agents—manogepix, rezafungin, ibrexafungerp, and olorofim—against 100 isolates of *Aspergillus section Terrei*, including amphotericin B (AmB)-non-wildtype and azole-resistant strains. These findings highlight the potential of novel antifungals, particularly olorofim, in addressing resistance in *Aspergillus section Terrei*, including azole- and AmB-resistant isolates. Further clinical studies are needed to validate their therapeutic roles and establish combination strategies for managing resistant infections.

**Initial Results of the International Efforts in Screening New Agents against Candida auris [Contributed Paper 9]**

*Candida auris* has emerged as a dangerous pathogen and scourge of hospitals and other clinical institutions. Resistance to currently available antifungal compounds is a problem this fungus presents. Given the challenges posed by *C. auris*, new antifungal therapies are urgently needed. Poester et al., in an international consortium, evaluated the in vitro activity of two agents, not currently in use, diphenyl diselenide (PhSe)2 and nikkomycin Z (nikZ), alone against *C. auris* and their interactions with existing antifungal drugs. The authors find that (PhSe)2 is not a viable candidate for treating *C. auris* owing to its lack of efficacy, and that nikZ is worthy of further exploration, especially in combination with echinocandins. These types of research collaborations are essential to finding new therapeutics against this pathogen.

**Severe Fungal Asthma: A Role for Biologics and Inhaled Antifungals [Contributed Paper 10]**

The excellent overview paper written by Moss explores the management of severe fungal asthma, which encompasses a spectrum of allergic fungal airway diseases, including allergic bronchopulmonary aspergillosis (ABPA) and severe asthma with fungal sensitization (SAFS). The current therapeutic armamentarium is reviewed. Advances in biologics and inhaled antifungals provide hope for improved management, but well-designed clinical trials are essential to validate their efficacy.

### 2.4. Immunology

Understanding the immunology of fungal infections is essential for understanding host–pathogen interactions and developing better therapeutic approaches. The following four papers [contributed papers 25–28] give an overview on some immunologic features of specific fungal infections:

**Vaccines to Prevent Coccidioidomycosis: A Gene-Deletion Mutant of *Coccidioides posadasii* as a Viable Candidate for Human Trials [Contributed Paper 11]**

Galgiani et al., the leading coccidioidomycosis vaccine research group, have brought forward several promising candidates as a vaccine. They present their latest and most exciting candidate to date. Coccidioidomycosis is a killer and appears to be expanding its range (likely related to climate change), and no currently available therapeutic has shown long-term, consistent prevention against recrudescences in infected persons. Environmental eradication of the fungus is not feasible. Natural infection with Coccidioides generally confers lifelong immunity, suggesting that a vaccine could be highly effective. However, no vaccine has been successfully developed due to technical, safety, and efficacy challenges. Their work provides a pathway to prevent this disease, and prevention offers what no other research avenue could contribute to eliminating this disease. This review summarizes the efforts carried out so far to develop a vaccine to prevent coccidiomycosis and reinforces that success with this vaccine could revitalize efforts to combat this neglected fungal disease and pave the way for future fungal vaccines.

**Allergic Bronchopulmonary Aspergillosis (ABPA) in the Era of Cystic Fibrosis Transmembrane Conductance Regulator (CFTR) Modulators [Contributed Paper 12]**

This review performed by Chatterjee et al. evaluates the impact of CFTR modulator therapies on ABPA, a serious complication in CF patients (CF-ABPA), focusing on modulator potential to mitigate inflammation, reduce fungal colonization, and improve clinical outcomes. CFTR modulator therapy, increasingly important in CF patient management, offers a promising approach to reducing the prevalence and severity of CF-ABPA. This extended review points to the need for further large-scale studies to understand modulator long-term effects on ABPA.

**Interplay of Cytokines and Chemokines in Aspergillosis [Contributed Paper 13]**

Shankar and co-authors reviewed the role of cytokines and chemokines in aspergillosis, focusing on pathogen recognition, immune responses, and their clinical implications for different forms of the disease, including allergic bronchopulmonary aspergillosis and invasive aspergillosis. The perspectives of both human and experimental infection are reviewed.

A balanced cytokine milieu is essential for controlling aspergillosis. Understanding these interactions can aid in developing targeted immunotherapies, particularly for immunocompromised patients. Therefore, advancing personalized immunotherapy could transform the management of aspergillosis, particularly for resistant and recurrent cases.

**Anticytokine Autoantibodies and Fungal Infections [Contributed Paper 14]**

Kappagoda and Deresinski review the role of anticytokine autoantibodies (ACAAs) in fungal infections. ACAAs disrupt cytokine pathways essential for immune defense, increasing susceptibility to fungal pathogens, representing an under-recognized cause of severe fungal infections in otherwise immunocompetent individuals. Examples from four different mycoses are discussed; innate immunity is reviewed. Improved diagnostic protocols and targeted immunomodulatory therapies are needed to address this emerging cause of secondary immunodeficiency.

### 2.5. Immunocompromised Patients and Associated Fungal Infections

The growing population of immunocompromised individuals, driven by advances in medical treatments and the increase in chronic diseases, has led to a proliferation in the prevalence and severity of fungal infections. Hence, the following three papers [contributed papers 29–31] discuss the complications of fungal infections in specific groups of immunocompromised patients:

**Invasive Aspergillosis after Renal Transplantation [Contributed Paper 15]**

Sigera and Denning provide a comprehensive review of the epidemiology, risk factors, diagnosis, clinical presentation, management, and prevention of invasive aspergillosis (IA) in renal transplant recipients. They show that the management of IA in renal transplant recipients requires a multidisciplinary approach, including prompt antifungal initiation, immunosuppression adjustments, and surgical considerations. One thing that distinguishes renal transplantation from other transplants is that surgeons can remove the kidney and stop immunosuppression; this is a difficult decision and peculiar to IA in this context. Early diagnosis and targeted therapy are crucial to mitigating the high mortality associated with this condition.

**Quantifying Deaths from Aspergillosis in HIV Positive People [Contributed Paper 16]**

Denning and Morgan have reviewed 747 articles on the mortality of Aspergillus infections in people infected with HIV, published over 36 years, and assessed data on 859 cases. This gives a comprehensive and global picture of the lethality of this opportunistic fungus in this population. The high mortality rate (83%) highlights the severity of the condition, despite advancements in HIV treatment, including antiretroviral therapy and antifungal medications like voriconazole. Improved diagnostic protocols and early treatment initiation are essential to reduce mortality. Aspergillosis in HIV patients is a subacute disease allowing time to make the diagnosis and intervene, given the mean time to death of 10 weeks. The lack of data from Africa suggests a research gap in regions with high HIV prevalence, potentially skewing the global understanding of the burden of aspergillosis. Enhanced surveillance and research in underrepresented regions are critical to addressing the global impact of aspergillosis in HIV/AIDS.

**Co-Occurrence of Gram-Negative Rods in Patients with Hematologic Malignancy and Sinopulmonary Mucormycosis [Contributed Paper 17]**

Egge et al. show that Gram-negative rods (GNRs) frequently coexist with Mucorales in sinopulmonary infections among patients with hematologic malignancies. GNRs may precede the fungal infection, or they may appear concomitantly. Treatment of GNRs may be a factor in the fungal emergence. Although co-occurrence does not worsen survival, further studies are needed to understand pathogen interactions and optimize therapeutic strategies.

## 3. Conclusions

We thank the eminent mycologists, all experienced authors and editors, who have contributed so much over the years to this discipline, for their excellent efforts in editing this Special Issue: Bertrand Dupont, Katsu Kamei, Rod Hay, and Karl Clemons. We thank the *Journal of Fungi*, a leading mycology journal, increasingly recognized, for suggesting this Special Issue, and its staff for their efforts in bringing this to fruition. We thank all the outstanding authors for their fine Contributions.

## References

[1] McKenna M. (2021). The deadly kingdom. Sci. Am. Mag..

[2] Buckley M., Buckley M. (2008). The Fungal Kingdom.

[3] Iliev I.D., Brown G.D., Bacher P., Gaffen S.L., Heitman J., Klein B.S., Lionakis M.S. (2024). Focus on fungi. Cell.

[4] Denning D.W. (2024). Global incidence and mortality of severe fungal disease. Lancet Infect. Dis..

[5] Biyun L., Yahui H., Yuanfang L., Xifeng G., Dao W. (2024). Risk factors for invasive fungal infections after haematopoietic stem cell transplantation: A systematic review and meta-analysis. Clin. Microbiol. Infect..

[6] Hennessee I., Benedict K., Bahr N.C., Lipner S.R., Gold J.A.W. (2024). Incidence and Risk Factors for Invasive Fungal Infections in Patients Initiating Tumor Necrosis Factor–Alpha Inhibitors for Inflammatory Bowel Disease and Rheumatoid Arthritis. Clin. Infect. Dis..

[7] Larcher R., Platon L., Amalric M., Brunot V., Besnard N., Benomar R., Daubin D., Ceballos P., Rispail P., Lachaud L. (2021). Emerging Invasive Fungal Infections in Critically Ill Patients: Incidence, Outcomes and Prognosis Factors, a Case-Control Study. J. Fungi.

[8] Salmanton-García J. (2024). Update on invasive fungal infections: Emerging trends in the incidence of fungal infections in immunosuppressed patients and associated conditions. Ther. Adv. Infect. Dis..

[9] Fisher M.C., Burnett F., Chandler C., Gow N.A.R., Gurr S., Hart A., Holmes A., May R.C., Quinn J., Soliman T. (2024). A one health roadmap towards understanding and mitigating emerging Fungal Antimicrobial Resistance: fAMR. NPJ Antimicrob. Resist..

[10] Seidel D., Wurster S., Jenks J.D., Sati H., Gangneux J.P., Egger M., Alastruey-Izquierdo A., Ford N.P., Chowdhary A., Sprute R. (2024). Impact of climate change and natural disasters on fungal infections. Lancet Microbe.

[11] De Gaetano S., Midiri A., Mancuso G., Avola M.G., Biondo C. (2024). Candida auris Outbreaks: Current Status and Future Perspectives. Microorganisms.

[12] American Society for Microbiology (2019). One Health: Fungal Pathogens of Humans, Animals, and Plants, Proceedings of the American Academy of Microbiology Colloquium, Washington, DC, USA, 18 October 2017.

[13] Woods M., McAlister J.A., Geddes-McAlister J. (2023). A One Health Approach to Overcoming Fungal Disease and Antifungal Resistance. WIREs Mech. Dis..

[14] WHO (2022). WHO Fungal Priority Pathogens List to Guide Research, Development and Public Health Action.

